# Role of tissue-type plasminogen activator and plasminogen activator inhibitor-1 in psychological stress and depression

**DOI:** 10.18632/oncotarget.19935

**Published:** 2017-08-04

**Authors:** Shih-Jen Tsai

**Affiliations:** ^1^ Department of Psychiatry, Taipei Veterans General Hospital, Taipei, Taiwan; ^2^ School of Medicine, National Yang-Ming University, Taipei, Taiwan

**Keywords:** brain-derived neurotrophic factor, tissue-type plasminogen activator, plasminogen activator inhibitor-1, major depressive disorder, stress

## Abstract

Major depressive disorder is a common illness worldwide, but the pathogenesis of the disorder remains incompletely understood. The tissue-type plasminogen activator-plasminogen proteolytic cascade is highly expressed in the brain regions involved in mood regulation and neuroplasticity. Accumulating evidence from animal and human studies suggests that tissue-type plasminogen activator and its chief inhibitor, plasminogen activator inhibitor-1, are related to stress reaction and depression. Furthermore, the neurotrophic hypothesis of depression postulates that compromised neurotrophin brain-derived neurotrophic factor (BDNF) function is directly involved in the pathophysiology of depression. In the brain, the proteolytic cleavage of proBDNF, a BDNF precursor, to mature BDNF through plasmin represents one mechanism that can change the direction of BDNF action. We also discuss the implications of tissue-type plasminogen activator and plasminogen activator inhibitor-1 alterations as biomarkers for major depressive disorder. Using drugs that increase tissue-type plasminogen activator or decrease plasminogen activator inhibitor-1 levels may open new avenues to develop conceptually novel therapeutic strategies for depression treatment.

## INTRODUCTION

Major depressive disorder (MDD) is a common mental disorder and is one of the leading causes of disability worldwide. Although the exact cause is unknown, several lines of evidences suggest that MDD in most people is caused by a combination of genes and stress, which can change brain chemistry and reduce the ability to maintain mood stability [1]. Genetic factors including genetic variants within genes that operate in stress response mediating neurobiological systems, neurotransmitters, and synaptic plasticity can increase susceptibility for MDD [2].

Synaptic plasticity allows changes in the connectivity between neurons and neuronal circuits permitting adjustment to environmental changes [3]. The mechanisms underlying these neuronal changes in response to either chronic or acute stress have been linked to the pathophysiology of major depression [4]. A group of proteins named neurotrophins are considered powerful molecular mediators in brain synaptic plasticity. Among them, brain-derived neurotrophic factor (BDNF) has key roles in the neurobiological mechanisms related to major depression [5].

BDNF, a small dimeric protein, is highly expressed in the hippocampus, prefrontal cortex, and amygdala, which are important areas for mood regulation [6]. BDNF can increase synaptic plasticity and its molecular mediators across multiple neurotransmitter systems [7]. Many animal and clinical studies suggest the importance of BDNF in MDD pathogenesis and therapeutic responses. For example, stress, a common precipitating factor for MDD, has been shown to decrease brain BDNF levels. The stress of immobilization can lower BDNF mRNA levels in the rodent hippocampus and other brain regions [8, 9]. In humans, stress at work reduced serum BDNF levels in hospital employees [10] and traumatic events have been found to be associated with lower BDNF plasma levels in patients with mood disorders [11]. In a clinical study, serum BDNF levels were significantly lower in MDD patients than in controls and depression severity mainly accounted for the negative correlation [12]. In a recent study, serum BDNF levels in patients with drug-naïve first-episode MDD were lower than those in the healthy controls [13]. In humans, postmortem studies have shown that brain BDNF was significantly downregulated at the RNA and protein levels in MDD subjects [14]. Some studies have associated a BDNF Val66Met functional polymorphism with MDD susceptibility [15, 16]. The role of BDNF in the pathophysiology of depression is further supported by the observation that the long-term administration of antidepressants in animals upregulates the production of brain BDNF [8]. Furthermore, an infusion of BDNF into the midbrain has an antidepressant-like influence in animal models of depression [17]. In a meta-analyses of eight studies comparing pre- and post-antidepressant treatment serum BDNF content, serum BDNF levels were elevated following antidepressant treatment [18].

These findings suggest that BDNF action could be impaired in MDD and that the repair of BDNF deficiency in response to antidepressant treatment may be a major part of the therapeutic mechanisms of antidepressants. However, not all studies support the proposition that decreased BDNF is a major cause of MDD. For example, although BDNF-deficient mice have lower brain BDNF levels than the wild type mice, BDNF-knockout mice did not show depressive behaviors [19]. Furthermore, the baseline BDNF mRNA levels did not differ in two genetic animal models of depression, one bred for helplessness in response to stress and the other bred for resistance to stress [20]. Clinical study by Hung et al. showed that serum BDNF levels presented no significant difference but the serum BDNF receptor (tyrosine kinase receptor B; TrkB) protein levels were significantly higher in MDD patients than in healthy controls [21]. Although treatment with major classes of antidepressants can increase peripheral BDNF levels [22], not all MDD patients benefit from antidepressant treatment, and the symptoms of depression occasionally deteriorate after antidepressant administration [23]. Thus, molecules other than BDNF that activate or inhibit BDNF function may involve in the pathogenesis of MDD.

Tissue-type plasminogen activator (tPA) is a proteolytic enzyme that activates plasminogen to plasmin. tPA is found not only in the blood, where its primary function is as a thrombolytic enzyme, but also in the brain, where it promotes neuronal synaptic plasticity [24]. The aim of this review is to summarize the current knowledge on the role of tPA -plasmin pathway in psychological stress and major depressive disorder. Furthermore, we discuss the development of potential therapeutic agents for major depression through modulating tPA and plasminogen activator inhibitor-1 (PAI-1).

### Brain tPA system

Plasmin is a proteolytic enzyme in the blood that causes fibrinolysis to prevent blood clots from growing. Plasminogen is the precursor to plasmin and can be converted to plasmin by tPA or the urokinase-type plasminogen activator [25]. Plasminogen activation is further controlled by specific plasminogen activator inhibitors that inhibit the effect of tPA. Among the plasminogen activator inhibitors, PAI-1 is the major endogenous inhibitor for tPA and has been implicated in a variety of thrombotic disorders [26]. The combination of PAI-1 and tPA terminates tPA enzymatic activity in the extracellular space.

The serine protease tPA is produced not only by endothelial cells but also by neurons and microglia of the central nervous system [27]. It is highly expressed in the brain and involves in processes such as learning and memory, stress reaction, neuronal degeneration, and addiction [27, 28]. In addition to tPA, other tPA-plasmin cascade components such as plasminogen and PAI-1 are also widely present in the brain [27].

### Plasmin and BDNF

**Figure 1:**
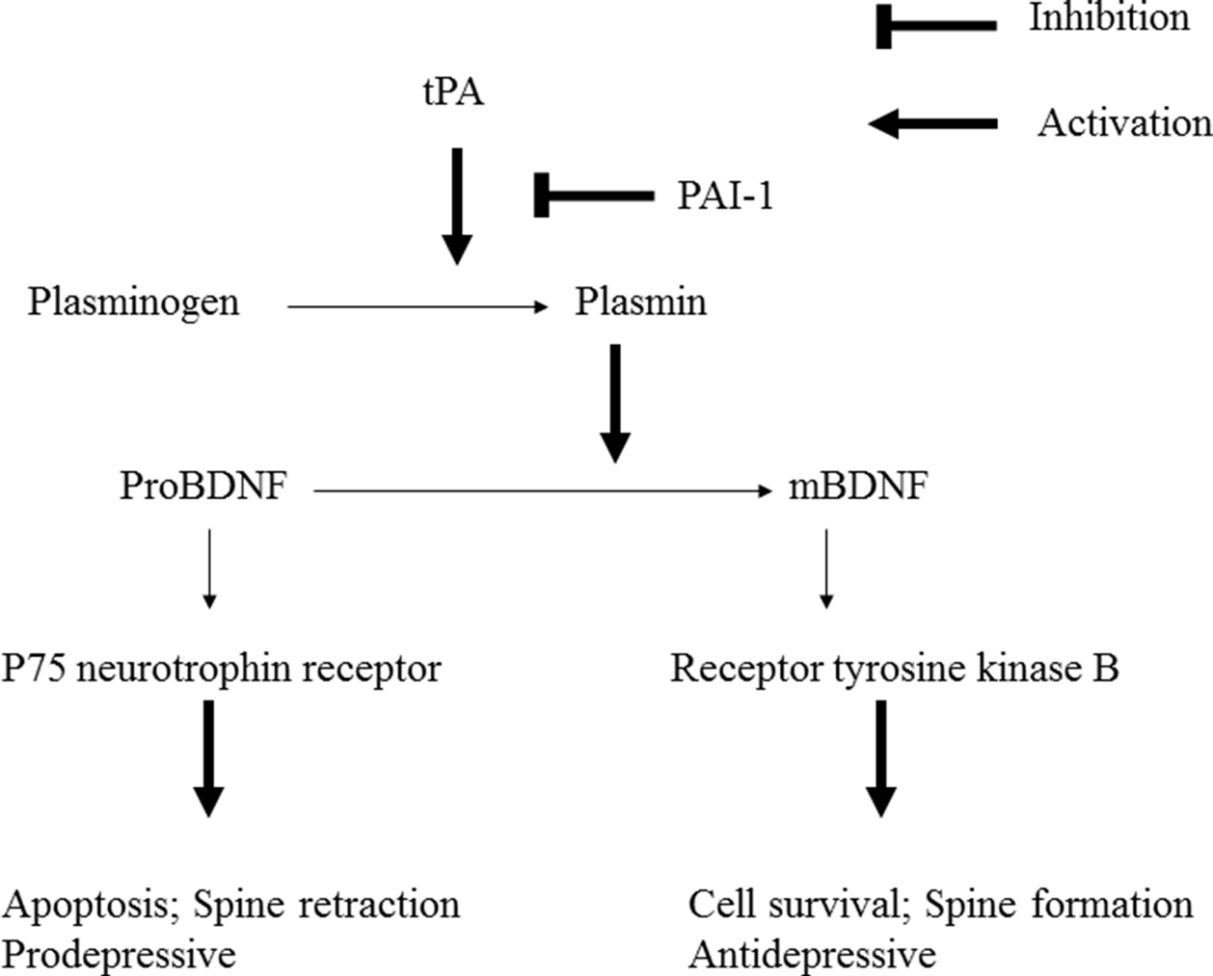
Plasminogen is activated to plasmin by tissue-type plasminogen activator (tPA) Plasminogen activator inhibitor-1 (PAI-1) is the major inhibitor of tPA. Plasmin can cleave the proBDNF to yield mature BDNF (mBDNF). Uncleaved proBDNF binds to the p75 neurotrophin receptor. mBDNF binds to receptor tyrosine kinase B.

BDNF is originally synthesized as the precursor protein proBDNF. In the secretion process, proBDNF can be converted to mature BDNF (mBDNF) by plasmin through the activation of plasminogen, which is dependent on tPA [29] (Figure 1). The functions of BDNF depend on its form (proBDNF or mBDNF) and the activation of two types of receptors. The p75 neurotrophin receptor (p75NTR) is a low affinity receptor for mBDNF and a high affinity receptor for the proBDNF [30]. The second receptor, neurotrophic tyrosine kinase receptor type 2 (NTRK2), also named TrkB, is a high affinity receptor for mBDNF. ProBDNF binding with high affinity to p75NTR leads to cell apoptosis, whereas mBDNF preferentially activates TrkB to bring about survival [31]. As proBDNF and mBDNF are assumed to elicit opposing biological effects [31], the regulation on the cleavage of proBDNF to mBDNF by plasmin becomes critical in the pathogenesis and therapeutic effects of major depression [32].

### BDNF, proBDNF and depression

The neurotrophin hypothesis of depression is based chiefly on animal study findings showing that decreased hippocampal BDNF levels are correlated with stress-induced depressive behaviors and that antidepressant administration increases the expression of BDNF [33]. However, increased levels of BDNF were found in some depression studies. For example, communal nest reared mice exhibited increased adulthood levels of BDNF and depressive behavior, which was displayed through higher levels of immobility during the forced swim test [34]. In an earlier study, we also found increased BDNF in the hippocampi of rats in an unpredictable chronic mild stress (UCMS) model of depression compared with control rats [35]. This inconsistency is because limited BDNF antibody specificity has prevented most studies that have investigated BDNF levels from distinguishing between proBDNF and mBDNF. In the brain, proBDNF and mBDNF activate two distinct receptors: p75NTR and TrkB. Therefore, methods that can effectively detect proBDNF and mBDNF are critical in discerning the potential depressive and antidepressant effects of BDNF [33].

In a learned helplessness (LH) model of depression, LH rats showed a reduction of mBDNF in the medial prefrontal cortex and a decline in CA3 and dentate gyrus of the hippocampus. Furthermore, levels of proBDNF were higher in the medial prefrontal cortex, but lower in the nucleus accumbens, of LH rats [36]. The finding that expression of proBDNF in the medial prefrontal cortex of LH rats was significantly higher than control rats was replicated by the same study group [37]. From these findings, it remains unclear whether these prodepressive effects result from a reduction in mBDNF levels or an accumulation of proBDNF. Using an UCMS paradigm, Bai et al. have similarly found that stress leads to increased levels of proBDNF, p75NTR, and sortilin but decreased levels of TrkB in the neocortex and hippocampus of rodent brains [38]. Furthermore, chronic administration of fluoxetine abolished this UCMS-induced upregulation of proBDNF. In that study, the injection of anti-proBDNF antibodies via intracerebroventricular and intraperitoneal routes into the UCMS rats significantly improved the stress-induced depressive behaviors and restored exploratory activity and spine growth [38]. Injection of adeno-associated-virus-proBDNF increased the depressive behaviors in naive rats, suggesting that proBDNF may also be implicated in the development of chronic stress-induced depressive behaviors [38]. Recent study also found UCMS caused a decrease in the ratio of BDNF/proBDNF in the hippocampus and injection of exogenous proBDNF into the CA1 region of rats caused depressive-like behaviors [39]. Another study found that injection of anti-proBDNF in anterior cingulate cortex reverses UCMS-induced depressive behaviors in mice [40].

In 2012, Yoshida et al. first demonstrated that serum levels of mBDNF, but not proBDNF, were significantly lower in patients with MDD than in control subjects [41]. This finding suggested that mBDNF may involve in the pathology of depression or as a biomarker for MDD. However, in another study of 40 drug-free patients with MDD and 50 healthy female controls, the protein and serum levels of proBDNF and p75NTR were higher in patients with MDD than in healthy controls, whereas mBDNF and TrkB levels were lower [42].

With the significant role of proBDNF in depression pathogenesis, tPA or other tPA/plasminogen cascade components such as PAI-1 could be important for depression pathogenesis or therapeutic mechanisms.

### Animal studies of tPA and PAI-1 in stress and depression animal models

Chronic exposure to stressful environments is a key risk factor contributing to the development of depression. Much of the current understanding about the pathogenesis of major depression has come from animal models comprising prolonged physical or social stress. In 2002, Yamamoto et al. demonstrated that PAI-1 is a major stress-regulated gene [43]. In their report, restraint stress led to a dramatic increase of plasma PAI-1 antigen and tissue PAI-1 mRNA with maximum induction in a tissue-specific and cell type-specific manner [43]. Similarly, after five weeks of chronic unexpected mild stress (CUMS) procedures, the expressions of PAI-1 and proBDNF increased in the prefrontal cortex and hippocampus of CUMS rats [44]. Since PAI-1 is the primary inhibitor of tPA, PAI-1 may decrease proBDNF cleavage and increase proBDNF levels by inhibiting the tPA–plasmin system.

Another rodent study suggested that brain tPA is critical for the development of stress-induced behaviors [45]. In that study, tPA was upregulated in the amygdala by acute restraint stress that preceded stress-induced increases of anxiety-like behaviors in mice and was subsequently inhibited by PAI-1. A study that revealed that tPA-knock down in the hippocampus induced depressive behaviors (increased immobility in the forced swim test and tail suspension test) further supported the influential role of tPA in depression [46]. These effects were reversed when tPA-over-expressing vectors were injected in the hippocampus. BDNF protein levels were simultaneously increased in the hippocampi of mice receiving tPA-expressing vectors [46].

### Human genetic studies of tPA and PAI-1 in depression

In humans, PAI-1 is encoded by the SERPINE1 gene. Our genetic association study demonstrated that the genetic variants of SERPINE1 gene may increase MDD susceptibility and the acute therapeutic response to selective serotonin reuptake inhibitors [47]. Another study found that the SERPINE1 gene promoter polymorphisms are involved in the antidepressant treatment of Alzheimer disease-related depression, without association with the depression susceptibility [48].

### Clinical studies of tPA and PAI-1 in depression

Associations between psychological factors, psychological stress, or psychiatric disorders and a state of hypercoagulability have been found in many studies [49].

Many clinical studies have investigated the relationship between the levels of plasma tPA and PAI-1 and psychological stress in MDD patients [50–64] (Table 1). The most consistent finding is elevated plasma PAI-1 levels during psychological stress or depression. The changes of tPA levels during stress reaction and depression are less consistent.

**Table 1:** Studies of peripheral tissue-type plasminogen activator (tPA) and plasminogen activator inhibitor-1 (PAI-1) levels to psychological stress and in MDD

Authors and Year of Publication	Study design and Subjects	Significant Findings	Adjusting confounders
Jern et al. (1994)	Mental stress (forced arithmetic) in 11 healthy young males	Plasma tPA levels increased significantly during stress.	None
Räikkönen et al. (1996)	Chronic stress in 69 healthy middle-aged men	Increased plasma PAI-1 levels.	age, smoking, alcohol consumption, physical activity, and metabolic factors
Ishizaki et al. (1996)	Psychosocial job stress in 213 middle aged male workers	High job demands were significantly related to decreases in tPA activity.	age, BMI, blood pressure, serum lipids, and smoking
Vrijkotte et al. (1999)	Work stress in 124 middle-aged, white-collar workers	Decreased tPA and increased PAI-1 plasma levels.	age, BMI, smoking habits, alcohol consumption, and physical activity
Hevey et al. (2000)	11 cardiac patients	Acute psychological stress decreased tPA and tPA/PAI-1 complexes.	None
von Känel et al. (2004)	48 community-dwelling elderly subjects	Mental stress elicited a decrease in tPA.	age, gender, BMI, blood pressure, serum lipids, and smoking
Eskandari et al. (2005)	45 premenopausal women with MDD vs 28 healthy controls	The MDD group exhibited statistically higher blood PAI-1 levels.	age, gender, BMI, and smoking
Lahlou-Laforet et al. (2006)	231 men (40 to 65 years old; 123 without coronary heart disease and 108 with coronary heart disease)	Plasma PAI-1 levels were higher in depressed subjects.	age, gender, BMI, blood pressure, serum lipids, and smoking
Matthews et al. (2007)	3292 community women	Higher depressive symptoms were related to higher plasma PAI-1 and tPA levels.	age, gender, BMI, blood pressure, physical activity, and menopausal status
Mausbach et al. (2008)	71 spousal dementia caregivers	Negative life events were positively associated with plasma PAI-1 concentrations in participants low in personal mastery but not in individuals high in personal mastery.	BMI, blood pressure, and metabolic factors
Geiser et al. (2008)	Anxiety patients (panic disorder with agoraphobia or social phobia) and control group (each n = 29)	Higher levels of PAI-1 in the patient group.	age, gender, BMI, blood pressure, smoking, alcohol consumption, physical activity, and metabolic factors
Pan et al. (2008)	3289 community residents aged 50-70	Depressive symptoms were not associated with plasma PAI-1 levels.	age, gender, BMI, blood pressure, smoking, alcohol consumption, physical activity, and metabolic factors
Shi et al. (2010)	24 late-onset geriatric MDD and 30 control subjects	Baseline plasma BDNF and tPA levels were significantly lower in MDD patients.	age, gender, and BMI
Smith et al. (2014)	18 male firefighters performed 18 min of simulated firefighting activities	tPA was enhanced immediately post firefighting but returned to baseline values by 2 h post firefighting. PAI-1 was depressed at 2 h post firefighting.	None
Malan et al. (2016)	181 black African urban-dwelling teachers (88 men, 93 women; aged 25-60 years)	In black men only, depressive symptoms were positively associated with plasma PAI-1 levels.	age, BMI, physical activity, blood pressure, and serum lipids

BMI: body mass index

### Depression treatment, tPA, and PAI-1

Studies have tested PAI-1 and tPA levels after depression treatment, medication and non-medication, and their results may further support the role of tPA system in MDD. In an animal study, after five weeks of CUMS to induce a depressive state, PAI-1 and proBDNF had increased in the prefrontal cortex and hippocampus [44]. In that study, the authors further investigated the effects of omega-3 polyunsaturated fatty acids (PUFAs) and sertraline (an antidepressant of the selective serotonin reuptake inhibitor) administration on brain tPA and PAI-1 levels in CUMS rats. Both agents were found to reverse the changes in behavioral tests and induce the expression of tPA in certain brain areas, but they failed to restore the CUMS-induced PAI-1 expression [44]. Moreover, sertraline treatment also accelerated the extracellular conversion of proBDNF into mBDNF in CUMS rats [44]. The later finding is in line with the report that, in human SH-SY5Y cells, treatment with different antidepressants significantly increased the production of mBDNF, but it decreased the production of proBDNF [65]. Similarly, another study found that the long-term administration of highly purified eicosapentaenoic acid, a specific omega-3 fatty acid, significantly and dose-dependently increased the plasma level of tPA and decreased PAI-1 level in rats [66]. A clinical trial in male cigarette smokers also demonstrated that omega-3 fatty acid supplementation increased plasma tPA concentrations [67].

Among human patients with anxiety and depression, Geiser et al., found lower PAI-1 in those given serotonergic antidepressants than in those without [68]. They further demonstrated that patients with anxiety and depression had lower PAI-1 after psychotherapy and improvement of psychiatric symptoms [68]. Recently, Jiang et al. examined serum protein concentrations in the tPA-BDNF pathway obtained from 35 drug-free depressed patients before and after antidepressant treatment and 35 healthy controls [69]. They found serum tPA and BDNF were significantly lower in the depressed patients than in controls, whereas proBDNF was higher. After 8 weeks of antidepressant treatment, tPA, BDNF and proBDNF levels were reversed.

Electroconvulsive therapy (ECT) is the most effective treatment for major depression. An animal study by Segawa et al. found that a single administration of electroconvulsive seizures (ECS) rapidly increased hippocampal levels of proBDNF and tPA, whereas rats receiving ten days of repeated ECS, accumulated proBDNF, which resulted in an increase in mBDNF levels [70]. In that study, chronic administration of imipramine (a tricyclic antidepressant) significantly increased mBDNF levels, but not proBDNF and tPA levels, suggesting that the therapeutic mechanism of imipramine differs from that of ECS [70].

Physical exercise has robust antidepressant properties. Mice with access to voluntary physical activity (not locked wheel exposure) for 28 days demonstrated a robust increase in hippocampal mBDNF protein levels and tPA mRNA expression; they also showed antidepressive effects compared with sedentary mice [71].

### Future research direction

### Plasma tPA and PAI-1 levels as potential MDD peripheral biomarkers

Diagnostic biomarkers are currently unavailable for MDD diagnosis and treatment, although easily accessible bodily fluids, including blood, are potential sources for the identification of biomarkers. Identification of biomarkers would aid in the diagnosis of MDD and in the selection of effective MDD medications [72]. Clinical studies have mostly showed increased blood PAI-1 levels during depressed or psychologically stressed states (Table 1). PAI-1 levels are affected by gender, age, body mass index, smoking status, and blood pressure. Therefore, these parameters should be introduced as adjusting variables in the analysis of PAI-1 levels in MDD study.

PAI-1 could be important in the cleavage of proBDNF to mBDNF. The ratio of mBDNF to proBDNF has been reported as a potential differential diagnostic biomarker for MDD and bipolar depression [73]. Previous studies (Table 1) have demonstrated that PAI-1 levels were more related to depressive mood than tPA levels were. Whether blood PAI-1 levels and the mBDNF to proBDNF ratio could be used as clinical biomarkers for MDD diagnosis warrants further exploration.

### Dysfunction of the tPA–plasmin pathway: a possible link between MDD and cardiovascular disease

MDD has been linked to a higher incidence of cardiac events in individuals with cardiovascular disease and in healthy populations [74–76]. Both psychological stress and depression are associated with an activation of coagulation and impairment of fibrinolysis. For example, high resolution two-dimensional Differential Gel Electrophoresis (2D-DIGE) proteome analysis on the platelet proteins found that the levels of fibrinogen beta chain and fibrinogen gamma chain were lower in patients with major depression than in healthy controls [77–79]. While analyzing the plasma proteome collected from MDD patients before the initiation of antidepressant medication, Martins-de-Souza et al. demonstrated high plasma fibrinogen levels are associated with a poor antidepressant response [80].

Hemostatic factors associated with the development of cardiovascular disease include fibrinogen, von Willebrand factor, tPA and PAI-1 [81]. Increased PAI-1 activity is associated with an increased risk of ischemic cardiovascular disease [82]. In addition, evidence from preclinical and clinical studies suggests that tPA and its inhibitor, PAI-1, are related to stress reaction and depression and are important fibrinolytic components. Therefore, dysfunction of the tPA–plasmin pathway could be a link between MDD and cardiovascular disease [83, 84].

### Manipulating tPA and PAI-1 levels for MDD treatment

According to the neurotrophic hypothesis of MDD, restoration of BDNF-TrkB function is the critical action of effective antidepressive treatments, including antidepressants and ECT. Since PAI-1 and tPA may involve in the transformation of proBDNF to mBDNF, tPA over-expression or a decrease in PAI-1 may present therapeutic effects for the treatment of depression especially for patients that have an abnormality in the tPA–plasmin pathway or comorbidities relating to cardiovascular disease. Several studies have reported that medication, ECS, and nutritional supplements may decrease PAI-1 or increase tPA levels.

An animal study demonstrated that omega-3 PUFAs and sertraline administration may induce the expression of tPA and facilitate the transformation of proBDNF to mBDNF in the prefrontal cortex and hippocampus of CUMS rats [44].

Hydroxymethylglutaryl coenzyme A reductase inhibitors, collectively known as statins, are a class of lipid-lowering medications. Some reports have indicated that statin treatment is associated with a reduced risk of depression [85, 86], although the mechanism underlying this antidepressant effect is unknown. *In vitro* studies have demonstrated that statins (fluvastatin or atorvastatin) inhibit PAI-1 and induce tPA in cultured cells [87, 88]. On the basis of these findings, researchers have postulated that statins may increase the transformation of proBDNF to mBDNF through activation of the tPA–plasmin pathway, thus causing an antidepressive effect [89].

An animal study using UCMS found that depression resulted in an increased expression of PAI-1 and upregulation of the proBDNF to mBDNF ratio, together with a decreased level of tPA [90]. The proBDNF to mBDNF ratio was further upregulated after ECS treatment in depressive rats [90]. Propofol alone did not reverse the changes in depressive rats, but when coadministered with ECS, it downregulated the proBDNF to mBDNF ratio, and increased tPA expression. The results of this study suggest that propofol combined with ECT may decrease the proBDNF to mBDNF ratio by increasing tPA expression [90].

Researchers studied a two-week fiber supplement in eleven healthy volunteer subjects who were instructed to consume oat husk tablets in addition to their regular diets [91]. Compared to the baseline, oat husk supplementation resulted in a reduction of plasma PAI-1 activity, and a six week washout period returned the PAI-1 activity level to the baseline. Thus, whether oat husk supplement has an antidepressive effect warrants further exploration.

### Other mental disorders, tPA, and PAI-1

Previous studies have found altered tPA and PAI-1 levels in psychological stress and MDD. Changes in tPA and PAI-1 levels have also been reported in schizophrenia [92, 93], addiction [94], and in disorders characterized by learning and memory deficits and by neuronal deterioration, such as Alzheimer disease [95]. Therefore, the dysfunction of the tPA-plasmin system might represent a common pathophysiological mechanism shared by several mental diseases.

### Limitations

Current major limitation in our understanding of the tPA-plasmin system and BDNF in depression is the lack of larger clinical depression studies that investigate the association between PA, PAI-1, plasmin, proBDNF and mBDNF. In addition, the function of the tPA-plasmin system is largely determined by genetic factors [96]. Genetic studies (e.g., mutation analysis, microarray, or genetic association studies) of genes related to the tPA-plasmin system in MDD are rare and the small sample sizes of these studies may limit statistical power.

## CONCLUSIONS

Synaptic plasticity and proBDNF cleavage, which are mediated by tPA, are crucial processes for mood regulation. Because the etiology of MDD is heterogeneous, the hypothesis that tPA–PAI-1 dysfunction might explain some MDD pathophysiology is supported by the animal studies, plasma level changes in humans under stress or depression, human genetic studies, and antidepressive treatment. The enhancement of PAI-1 levels seems to be a common finding under stressed or depressed states, and it may be a candidate for a biological marker for depression. Additional studies are needed to determine how interventions aiming specifically at correcting activity of tPA or PAI-1 may contribute to novel strategies for the management of this common mental disorder.
